# Clinical impact of tropism testing in a real-life cohort of HIV infected patients: a retrospective observational study

**DOI:** 10.1186/s12879-019-4047-7

**Published:** 2019-05-24

**Authors:** Laurène Deconinck, Olivier Robineau, Michel Valette, Philippe Choisy, Laurence Bocket, Agnes Meybeck, Faiza Ajana

**Affiliations:** 1 Infectious Diseases Department, Tourcoing Hospital, Tourcoing, France; 2 Virology Department, Lille University Hospital, Lille, France

**Keywords:** HIV, Viral tropism, CCR5 receptor antagonists, Maraviroc, Practice studies, Real-life management

## Abstract

**Background:**

The circumstances of prescription of tropism tests clinically relevant in treatment-experienced patients are unclear.

**Methods:**

We performed a monocentric retrospective analysis of all tropism tests performed between 2006 and 2015 in HIV-infected patients on antiretroviral therapy (ART) without MVC. The motivation of tropism determination was collected. Factors associated with MVC prescription were determined using logistic regression analysis.

**Results:**

Five hundred sixty-three tests were performed in experienced patients not receiving MVC. Reasons for tropism performance were: virological failure (44%), side effects or drug-interactions (37%), simplification or sparing strategies (11%), immunological failure (5%), and improvement of neurological diffusion (3%). MVC was prescribed in 110 cases (20%), though 366 tests (65%) revealed a tropism CCR5. MVC was more often prescribed before 2011 (OR 3.65, 95% CI 2.17–6.13) and in patients with multiple previous ART regimens (less than 4 ART regimens compare to more than 10 ART regimens (OR 0.34, 95% CI 0.15–0.74)).

**Conclusions:**

In experienced patients not receiving MVC, tropism test prescription should be restricted to patients with virological failure and limited therapeutic options such as patients already treated with a wide range of ART regimens.

## Background

To enter the host cell, the Human Immunodeficiency Virus type 1 (HIV-1) binds to the cellular receptor CD4 and one of the cellular co receptors CCR5 or CXCR4. Maraviroc (MVC) is a first-in-class selective CCR5 antagonist. It is also the first host-targeted antiretroviral drug. Next to its efficacy in suppressing plasma HIV-RNA, it has been hypothesized that it could have immunomodulatory effects [1]. Furthermore, several reports suggest that MVC might have beneficial effects on the inflammatory component of HIV and John Cunningham (JC) virus-associated central nervous system disease [2]. MVC has been registered for the treatment of antiretroviral therapy (ART)-naive (USA only) and ART-experienced HIV-1 infected patients [3]. Prior to its prescription, HIV-1 tropism should be determined, as the drug is ineffective against CXCR4-tropic HIV. European AIDS Clinical Society (EACS) guidelines and french experts recommend that tropism testing should be performed if considering MVC prescription [4–6]. Since its first use in salvage therapy, MVC has been evaluated in various clinical situations such as switch or nucleoside reverse transcriptase inhibitors (NRTI)-sparing strategies [7–9]. The potential extension of the indications of MVC could generate an increase in prescriptions of tropism determinations. To define the circumstances of prescriptions for which tropism testing is clinically relevant, we reviewed the indications and clinical impact of tropism tests performed in ART-experienced patients followed in our center.

## Methods

### Patients and hospital setting

We performed a retrospective analysis of all phenotypic and genotypic tropism tests performed in our centre between March 2006 and July 2015. Tropism has been determined by Trofile assay, or genotypic sequencing in HIV-RNA or in proviral HIV-1 DNA in PBMCs. Tropisms prescribed within the framework of a clinical trial, in treatment-naive patients and in patients already receiving MVC were excluded. Only tropisms performed in treatment-experienced HIV infected patients not receiving MVC were included. All patients have signed an agreement allowing the record of their data in a database (NADIS) approved by a French data protection authority (Commission nationale de l’informatique et des libertés (CNIL) 770,134) [10]. As a retrospective survey and in accordance with the European General Data Protection Regulation (n^o^ 2016/679), this study has been locally registered in Dron Hospital. Furthermore, local ethical committee has approved this research (comité d’éthique du Centre Hospitalier Gustave Dron).

### Data collection

Demographic data and characteristics of patients were collected. Following data on HIV infection at the time of MVC tropism performance were recorded: CD4 cell count and plasma HIV viral load, HIV disease staging according to CDC Classification System for HIV-Infected Adults and history of antiretroviral therapy (number of successive antiretroviral regimens and their duration, resistance and ART at the time of tropism performance). The justification of tropism performance was specified by means of a questionnaire sent to the infectious disease physician in charge of each patient. Motivation of tropism determination was specified: virological failure, side effects or drug-interactions, simplification or sparing strategies, improvement of neurological diffusion or immunological failure. Tropism determination for improvement of neurological diffusion and immunological failure were considered as prescription for specific MVC properties.

### Statistical analysis

First, a descriptive analysis of the overall population at the time of tropism performance was conducted. Patients with CCR5 tropism receiving MVC and those not receiving MVC were described and compared. Continuous variables were expressed as mean and standard deviation. They were compared using the Mann-Whitney test. Categorical variables were expressed as number and percentage. They were compared using the Fisher’s exact test. Differences between groups were considered to be significant for variables yielding a *p* value < 0.05.

Second, to determine the independent effect of the variables on the prescription of MVC, we performed a logistic regression analysis using the purposeful selection of covariates. All covariates with *p* < 0.2 in the unadjusted model were entered into the multivariate model.

Last, we made the assumption that reason associated with prescription of MVC might change over time. Thus, we performed a subgroup analysis dividing the tropisms in two groups defined by their date of performance, from 2006 to 2010 and from 2011 to 2015.

All statistical analyses were performed using Stata software version 7.0.

## Results

### Study population

**Fig. 1:**
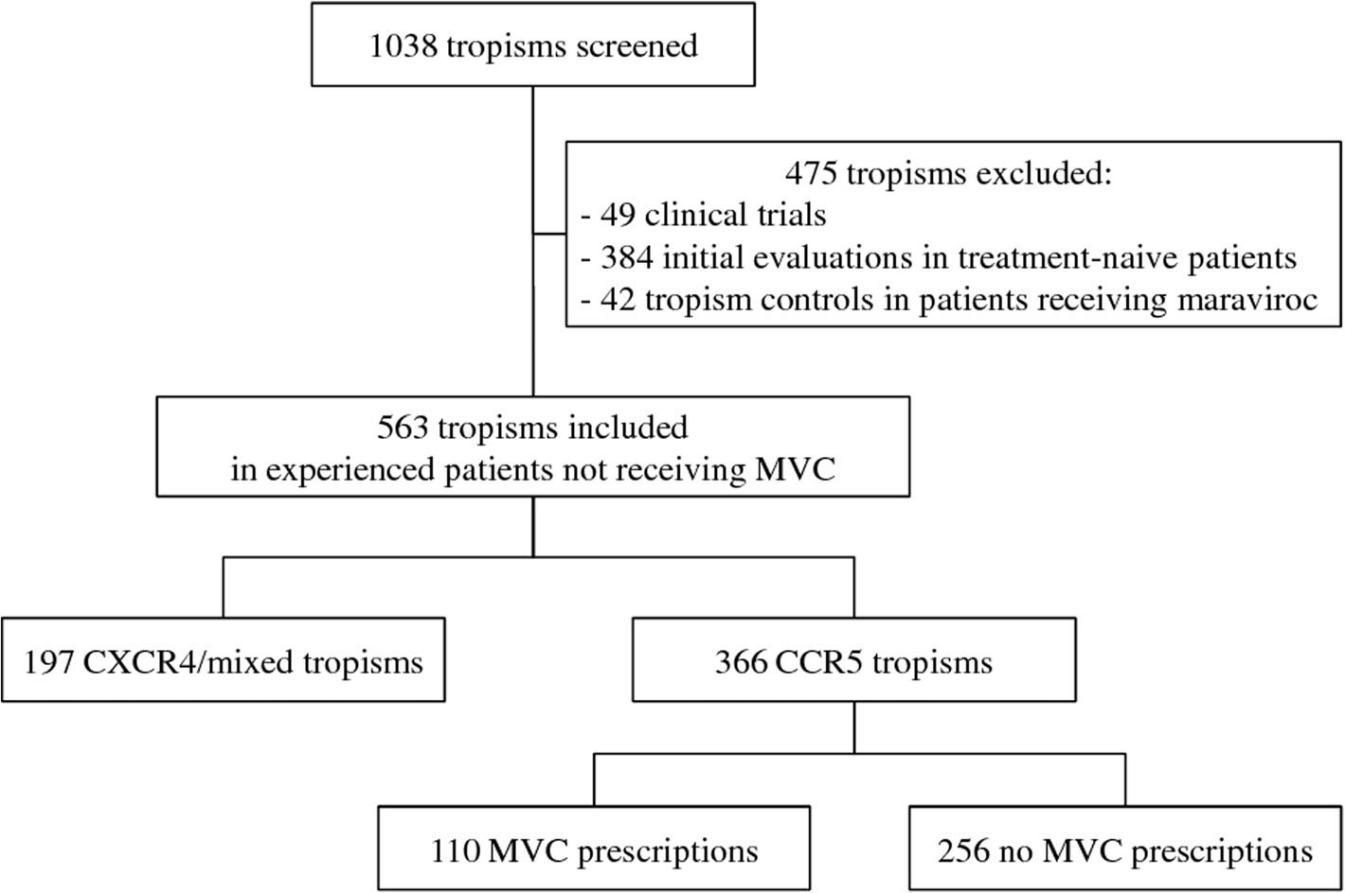
Flow diagram of tropisms included in the study

From 2006 to 2015, a total of 1038 tropism tests were performed (Fig. 1). Four hundred seventy-five tropisms were excluded: 49 tropisms prescribed within the framework of a clinical trial, 384 prescribed as part of the initial evaluation in ART naive patients and 42 to control tropism in patients treated with MVC containing regimen. Five hundred sixty-three tests prescribed in experienced patients not receiving MVC were included in the study.

**Fig. 2:**
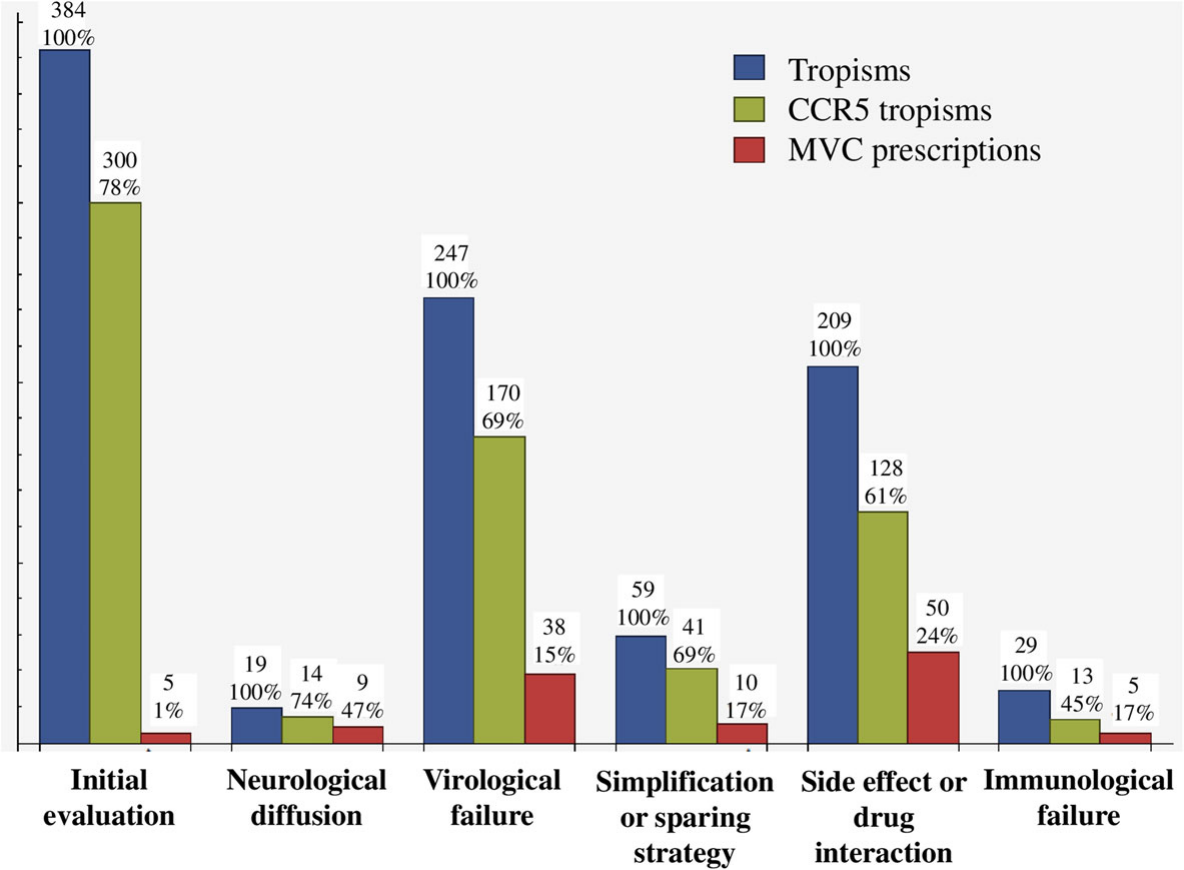
Tropism tests, CCR5 tropisms and MVC prescriptions depending on reasons for tropism performance

At the time of tropism performance, the mean CD4 count was 503/mm3; the CDC stage was A in 271 cases (48%), B in 147 cases (26%) and C in 145 cases (26%); the viral load was not undetectable in 305 cases (54%). The mean time between ART initiation and tropism was 10 years, with a mean of 6 previous ART regimens; the virus was resistant to a mean of 1 ART family. Reasons for tropism performance were: virological failure in 247 cases (44%), side effects or drug-interactions with the actual regimen in 209 cases (37%), simplification or sparing strategies in 59 cases (11%), immunological failure in 29 cases (5%), and improvement of neurological diffusion in 19 cases (3%) (Fig. 2). Tropism was CCR5 in 366 cases (65%).

### Population with CCR5 tropism

Characteristics at the time of test performance of the 366 patients with CCR5 tropism are described in Table 1. Tests were prescribed before 2011 and after 2011 in 164 (45%) and 202 (55%) cases respectively. ART regimen was 2 NRTI and 1 PI in 127 cases (35%), 2 NRTI and 1 NNRTI in 66 cases (18%), 2 NRTI and 1 integrase inhibitor in 22 cases (6%), PI monotherapy in 19 cases (5%), other regimens in 88 cases (24%) and no ART in 44 cases (12%). Reasons for tropism performance were: virological failure in 170 cases (46%), side effects or drug-interactions with the actual regimen in 128 cases (35%), simplification or sparing strategies in 41 cases (11%), immunological failure in 13 cases (4%), and improvement of neurological diffusion in 14 cases (4%). Tropism was prescribed for a specific MVC property in 27 cases (7%). MVC was finally prescribed in 110 cases (30%). The mean time between tropism and MVC introduction was 89 days. Six months after MVC introduction, the viral load was undetectable for 68% of patients and there was an increase of more than 10% of the CD4 count in 49% of patients. Concerning the 256 individuals that finally not received MVC despite a CCR5 tropism, 39% stayed under the treatment association that preceded tropism testing, 22% changed for a regimen including an integrase inhibitor and 39% changed for other associations including a protease inhibitor as third agent.

**Table 1:** Characteristics of experienced patients not receiving MVC at the time of tropism test performance

	Total (*n* = 366)	MVC prescription (*n* = 110)	No MVC prescription (*n* = 256)	*p*
Test performance before 2011	164 (44.8)	75 (68.2)	89 (34.8)	< 0.01
Age (years)	47 (10.9)	49 (10)	47 (11.3)	0.05
Male sex	263 (71.9)	80 (72.7)	183 (71.5)	0.81
Risk group				0.13
* Heterosexual*	175 (47.8)	50 (45.5)	125 (48.8)	
* Homosexual*	145 (39.6)	50 (45.5)	95 (37.1)	
* Other*	46 (12.6)	10 (9.1)	36 (14.1)	
CDC stage				0.03
* A*	185 (50.6)	46 (41.8)	139 (54.3)	
* B*	84 (22.9)	34 (30.9)	50 (19.5)	
* C*	97 (26.5)	30 (27.3)	67 (26.2)	
CD4 count (/mm3)	515.9 (286.5)	539.7 (262.5)	505.8 (295.9)	0.27
Undetectable viral load	155 (42.3)	52 (33.5)	103 (66.5)	0.21
Time between ART initiation and tropism (years)	9.9 (6.1)	10.9 (6)	9.4 (6.1)	0.03
Time between last ART change and tropism (years)	2.2 (2.2)	2.4 (2)	2.1 (2.3)	0.09
Number of previous ART regimens	6 (5.3)	7.9 (7.1)	5.2 (4)	< 0.01
Number of ART families with resistance	1.1 (1)	1.2 (1.1)	1.1 (0.9)	0.23
Reason for tropism performance				< 0.01
* Virological failure*	170 (46.4)	38 (34.5)	132 (51.6)	
* Side effect or interaction*	128 (35)	50 (45.5)	78 (30.5)	
* Neurological diffusion*	14 (3.8)	7 (6.4)	7 (2.7)	
* Sparing strategy or simplification*	41 (11.2)	10 (9.1)	31 (12.1)	
* Immunological failure*	13 (3.6)	5 (4.6)	8 (3.1)	

Mean (standard deviation) and number (%)

*MVC* Maraviroc, *ART* Anti Retroviral Therapy

### Factors associated with prescription of MVC

In univariate analysis, MVC prescription was significantly associated with period of prescription (*p* < 0.01), age at the time of test (*p* = 0.05), CDC stage (*p* = 0.03), time from ART initiation (p = 0.03), number of previous ART regimens (*p* < 0.01), and reason for tropism performance (*p* < 0.01) (see Table 1).

In multivariate analysis, MVC was more often prescribed after a tropism test performed before 2011 (OR 3.65, 95% CI 2.17–6.13) and in patients with multiple previous ART regimens (less than 4 ART regimens (OR 0.34, 95% CI 0.15–0.74), 4 to 5 ART regimens (OR 0.32, 95% CI 0.14–0.74), 6 to 10 ART regimens (OR 0.46, 95% CI 0.22–0.97) compare to more than 10 ART regimens). Tropism prescription for virological failure was not associated with MVC prescription (OR 1.10, 95% CI 0.62–1.98) (see Table 2).

**Table 2:** Factors associated with MVC prescription after a tropism test

	Factors associated with MVC prescription			
	Univariate		Multivariate	
	OR (CI 95%)	*p*	OR (CI 95%)	*p*
Period of test:				
*Before 2011*	4.16 (2.51–6.89)	< 0.01	3.65 (2.17–6.13)	< 0.01
*After 2011*	1		1	
Age (years):		0.26		
*< 35*	0.60 (0.25–1.45)			
*35–43*	0.68 (0.35–1.32)			
*44–51*	1.1 (0.63–1.93)			
*> 51*	1			
Time between ART initiation and tropism (years):		0.03		
*< 5*	0.50 (0.27–0.92)			
*5–10*	0.88 (0.49–1.58)			
*> 10*	1			
Number of previous ART regimens:		< 0.01		
*< 4*	0.24 (0.11–0.51)		0.34 (0.15–0.74)	< 0.01
*4–5*	0.25 (0.11–0.55)		0.32 (0.14–0.74)	< 0.01
*6–10*	0.39 (0.19–0.79)		0.46 (0.22–0.97)	0.04
*> 10*	1		1	
CD4 count (/mm3):		0.02		
*≤200*	0.34 (0.13–0.85)			
*201–500*	0.86 (0.52–1.42)			
*> 500*	1			
Reason for tropism performance:				
*Virological failure*	1.95 (1.20–3.16)	< 0.01	1.10 (0.62–1.98)	0.73
*Other*	1		1	

*MVC* Maraviroc, *ART* Anti Retroviral Therapy

### Subgroup analysis depending on the period of tropism test performance

**Fig. 3:**
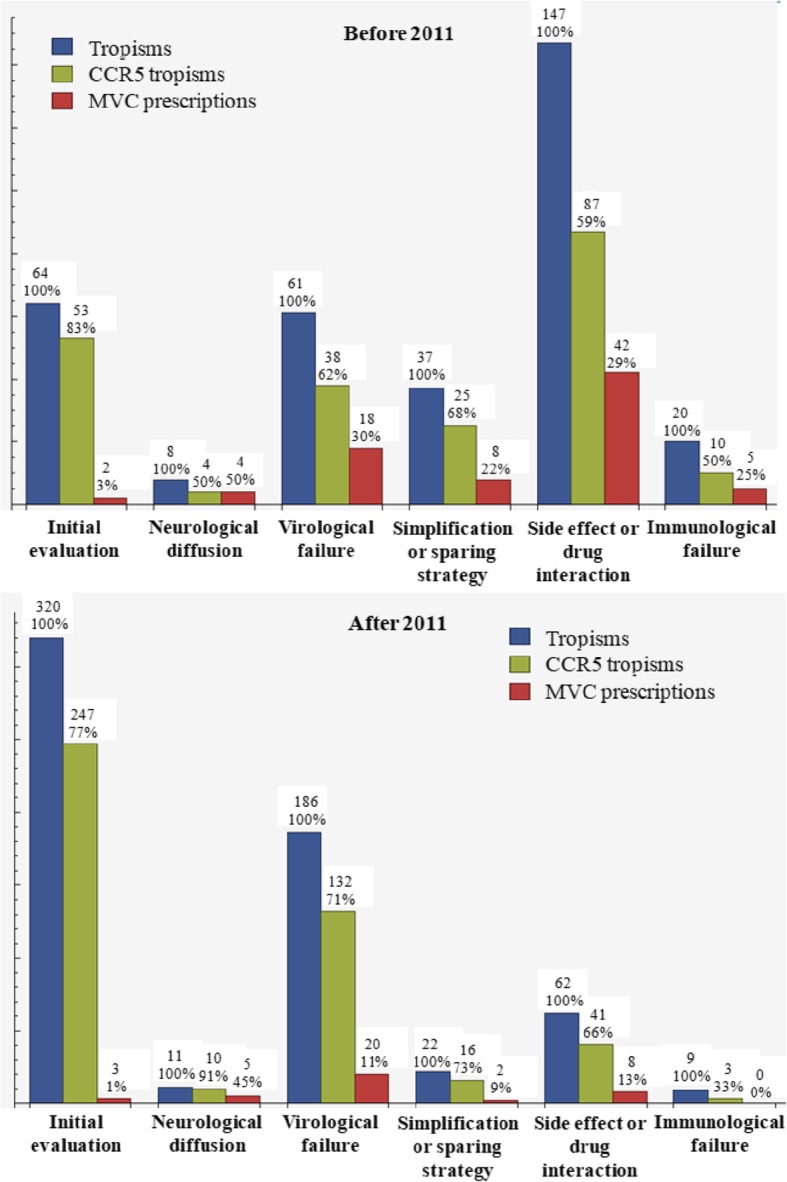
Tropism tests, CCR5 tropisms and MVC prescriptions depending on reasons for tropism performance before and after 2011

Reasons for tropism prescription and proportions of MVC introduction before and after 2011 are reported in Fig. 3. For tropism tests performed during the first period, multivariate analysis revealed that MVC was more often prescribed in patients with multiple previous ART regimens (less than 4 ART regimens (OR 0.24, 95% CI 0.06–0.94), 4 to 5 ART regimens (OR 0.25, 95% CI 0.08–0.84), 6 to 10 ART regimens (OR 0.35, 95% CI 0.13–0.95) compare to more than 10 ART regimens). There was no association with the reason for tropism prescription. For tropism tests performed during the second period, multivariate analysis showed no factor significantly associated with MVC prescription. Nevertheless, MVC prescriptions tended to be more frequent after a test performed for specific MVC properties (test prescribed for non-specific MVC properties compare to specific MVC properties: OR 0.23, 95% CI 0.05–1.15, *p* = 0.07), without reaching significance.

## Discussion

This study was an opportunity to analyze reason of tropism prescription and its effect on treatment strategies in ART-experienced patients. The major outcome of this work is that only 20% of all tropism prescriptions lead to MVC initiation. This work also suggests that reason to finally prescribe MVC might change over time.

In our cohort, a CCR5 tropism was found in 65% of cases, which is consistent with the literature [11, 12]. Among these tests, MVC was prescribed in only one third of cases. In a retrospective cohort study conducted in New-York City, McCarthy et al. reported that 10% of tests showing CCR5 tropism were followed by MVC prescription [13]. Comparable results were obtained by Wyatt et al. after reviewing all tropism tests performed in their referral centre in London, with 18% of patients eligible for MVC receiving it following tropism determination [14]. Our results confirm this low proportion of MCV prescription following CCR5 tropism and a need for a more focused prescriptions of tropism test in experienced patients.

This work also describes reason for CCR5 tropism prescription. MVC has been shown to be of interest in various clinical situations in experienced patients. It has been evaluated in patients with virological failure, and in patients with poor tolerability of NRTI, NNRTI or PI in sparing strategies [3, 7, 8, 15–18]. Studies have also reported a specific interest in patients with poor immune restoration, with greater increase in CD4 T-cells [19, 20]. Some other studies have reported that MVC can be effective in patients with neurological involvement [16]. Guidelines are not very restrictive. EACS guidelines suggests undertaking tropism testing if use of CCR5 antagonist is considered in patients who fail treatment, who have toxicity of current treatment, or who suffer from central nervous system pathology [4]. In our study population, virological failure was the main reason of tropism performance. The second reason was side effects or drug-interactions with the current regimen. Only a few tests were prescribed for a specific MVC property such as immunological failure or improvement of neurological diffusion.

Characteristics of patients who finally benefit of MVC prescription after a tropism test in real life setting are not well known. This work is the first specifically designed to determine why MVC is finally prescribed. We found that the number of previous ART regimens and the period of tropism prescription were associated with MVC prescription. MVC was more often prescribed in experienced patients already treated with multiple ART regimens. Indeed, for these patients, because of resistance and previous drug toxicities, MVC remains one of the last therapeutic options available. Similarly, in his retrospective study analyzing the use of tropism test in clinical practice, McCarthy suggests that tropism tests should only be performed in case of virological failure after a genotype revealing resistance mutations limiting the therapeutic options [13]. However, in his cohort, virological failure was often due to poor adherence to treatment and CCR5 tropisms were not followed by MVC prescription in these cases. Wyatt has also evaluated utility of tropism tests and confirmed that presence of other ART resistance increased the likelihood of patients starting MVC after tropism test performance [14]. In our cohort, resistance alone was not associated to MVC prescription, indicating that drug toxicities might also play a role for the choice of MVC containing regimen in experienced patients already treated with numerous antiretroviral regimens.

The other factor significantly associated with MVC prescription was the period of tropism test performance. MVC was more often prescribed after a tropism test performed before 2011. This can be explained by the attraction of a new drug during the first years after its release. It can also be due to the fact that MVC appeared disappointing after a few years [1, 21]. Indeed, MVC and raltegravir, the first integrase inhibitor, were released simultaneously. Raltegravir, and more recently dolutegravir, have shown great efficacy in case of multi-drug resistance [22, 23], and a good tolerance profile. Thus, integrase inhibitors have rapidly been preferred. In our cohort, subgroup analysis showed that before 2011 number of previous ART regimens was significantly associated with prescription of MVC, while after 2011 tropism test determination for specific MVC properties tends to be more often followed by MVC prescription. Our results reflect the change of clinical practice, with actual prescription of MVC only in cases for which a specific property of MVC is interesting. Likewise, Llibre et al. conducted a multicentre retrospective study assessing conditions of MVC prescription between 2012 and 2013. MVC was used in salvage therapy only in half of the cases [15]. Thus, prescription of tropism test should be adapted to the evolution of MVC prescription habits.

Nowadays, tropism test prescription should be restricted to patients with not only virological failure but also limited therapeutic options such as patients already treated with a wide range of antiretroviral regimens. Tropism test could also remain interesting in patients for who a specific property of MVC is expected such as neurological diffusion or immunomodulatory effect.

Our study has several limits associated to the retrospective data collection. Moreover, it is a monocentric study with a limited number of prescribers. Our findings might not be generalized as clinical practice may differ across institutions and countries.

## Conclusion

Tropism test is often performed in patients with virological failure but rarely leads to the prescription of maraviroc. At a time when the available treatments are numerous, only tropism prescription in patients with virological failure and limited therapeutic options seems to remain clinically relevant.

## Abbreviations

ART: Antiretroviral therapy
CDC: Centre for Disease Control
EACS: European AIDS Clinical Society
HIV: Human immunodeficiency virus
MVC: Maraviroc
NNRTI: Non nucleoside reverse transcriptase inhibitor
NRTI: Nucleoside reverse transcriptase inhibitor
PBMC: Peripheral blood mononuclear cell
PI: Protease inhibitor
USA: United States of America
